# Population variation in the trophic niche of the Trinidadian guppy from different predation regimes

**DOI:** 10.1038/s41598-017-06163-6

**Published:** 2017-07-18

**Authors:** Eugenia Zandonà, Christopher M. Dalton, Rana W. El-Sabaawi, Jason L. Howard, Michael C. Marshall, Susan S. Kilham, David N. Reznick, Joseph Travis, Tyler J. Kohler, Alexander S. Flecker, Steven A. Thomas, Catherine M. Pringle

**Affiliations:** 1 Department of Biology, Drexel University, Philadelphia, PA 19104 USA; 2 Ecology and Evolutionary Biology, Cornell University, Ithaca, NY 14853 USA; 3 Odum School of Ecology, University of Georgia, Athens, GA 30602 USA; 4 Department of Biology, University of California, Riverside, CA 92521 USA; 5 Department of Biological Science, Florida State University, Tallahassee, FL 32306 USA; 6 School of Natural Resources, University of Nebraska, Lincoln, NE 68583 USA; 7 Department of Ecology – IBRAG, Universidade do Estado do, Rio de Janeiro, RJ 20550-013 Brazil; 8 Department of Biology, University of Victoria, PO Box 1700 Station CSC, Victoria, BC V8W 2Y2 Canada; 9 Department of Biological Sciences, Florida International University, Miami, FL 33199 USA; 10 Faculty of Science, Department of Ecology, Charles University in Prague, Viničná 7, Prague, 2 - 128 44 Czech Republic

## Abstract

Population variation in trophic niche is widespread among organisms and is of increasing interest given its role in both speciation and adaptation to changing environments. Trinidadian guppies (*Poecilia reticulata*) inhabiting stream reaches with different predation regimes have rapidly evolved divergent life history traits. Here, we investigated the effects of both predation and resource availability on guppy trophic niches by evaluating their gut contents, resource standing stocks, and δ^15^N and δ^13^C stable isotopes across five streams during the wet season. We found that guppies from low predation (LP) sites had a consistently higher trophic position and proportion of invertebrates in their guts and assimilate less epilithon than guppies from high predation (HP) sites. Higher trophic position was also associated with lower benthic invertebrate availability. Our results suggest that LP guppies could be more efficient invertebrate consumers, possibly as an evolutionary response to greater intraspecific competition for higher quality food. This may be intensified by seasonality, as wet season conditions can alter resource availability, feeding rates, and the intensity of intraspecific competition. Understanding how guppy diets vary among communities is critical to elucidating the role of niche shifts in mediating the link between environmental change and the evolution of life histories.

## Introduction

Trophic niche differentiation and resource polymorphisms are important and ubiquitous ecological phenomena^1, 2^, and are often the result of the exploitation of new resources or habitats in response to extrinsic environmental change^3^. Many of the best-characterized examples of trophic niche differentiation describe within-population differences (e.g. the benthic-limnetic trophic differentiations in many fish species)^4, 5^, but examples of such polymorphisms between populations inhabiting different types of environments are more scarce^2, 6, 7^. Individuals that are found in different habitats or that experience different levels of resources, predation, or competition can differ in morphology, diet selectivity, and feeding behaviour^6–8^. These trait differences have an adaptive significance, increasing the fitness of individuals in the habitats where they are found^9^. Because niche shifts may arise due to phenotypic plasticity or rapid evolution of relevant traits^5, 10^, understanding the ecological and evolutionary forces shaping and constraining niche breadth remains a relevant and important challenge for researchers and conservationists, especially in the face of rapid environmental change.

Here we assess the role of available food resources and predation pressure as drivers of intraspecific niche divergence between populations of the Trinidadian guppy (*Poecilia reticulata*), one of the most important model organisms for studying the links between ecological and evolutionary change. Guppies on the island of Trinidad have evolved divergent life history traits in response to abundance of predators, which is reduced at upstream reaches due to barrier waterfalls that excludes most predator species^11^. Guppies from sites with high predation intensity (hereafter HP sites) experience high extrinsic mortality and mature at an earlier age and smaller size, while allocating more energy to reproduction than guppies in sites with low predation intensity (hereafter LP sites)^11^. Adaptation of guppies to reduced predation intensity has occurred in multiple rivers that vary in environmental conditions and predator assemblages, making it a useful example of convergent evolution^12^.

The evolution of the LP phenotype in response to a reduction in predation pressure was initially attributed to differences in size-specific mortality rates between HP and LP populations^11^. However, differences in mortality alone do not explain the evolution of LP guppies^13, 14^. The lower predation intensity is associated with increased guppy population density and reduced food availability^14–16^. In addition to these differences, HP and LP locations often present broad environmental differences, which might significantly alter the resources available to guppies. HP sites - due to their downstream location - generally have greater light, higher primary productivity, and more resources per capita than LP sites^17^, and these differences can also be present within sites characterized by the same predation regime^18, 19^. Therefore, understanding how guppy diets vary among predation environments and across gradients in resources is critical to elucidating the role of niche shifts in mediating the link between environmental change and the evolution of life histories.

Previous research on guppy diets from a limited number of sites have resulted in some contradictory findings. Studies conducted in two streams during the dry season^20^ and in mesocosms^21^ showed that differences in guppy diet quality and selectivity are associated with differences in life history patterns. In these studies, HP fish fed more consistently on invertebrates and selectively fed on invertebrates of high nutritional quality. LP guppies were observed to have longer guts. These dietary differences were maintained between seasons^22^ and after guppies are transferred to the lab and offered the same diet^23^. Longer guts often represent an adaptation to low quality food^24, 25^, thus supporting the hypothesis that LP guppies are more herbivorous/detritivorous, feeding on lower quality resources. We refer to this as the “specialization” hypothesis, which suggests that HP guppies are more selective and specialized on higher quality resources (i.e. invertebrates).

In contrast, it is also possible that, due to high intraspecific competition for limited resources, LP guppies have evolved to feed on whatever food resources are available, be it limited high-quality invertebrates or abundant low-quality detrital and algal foods, as optimal foraging theory would predict^26^. In *ad libitum* feeding trials, where guppies were fed only one type of large invertebrate, LP guppies had higher consumption rates than HP guppies. LP trophic morphology also differed from HP, suggesting they are adapted to a different diet^27^. The results of this experiment support an “efficiency” hypothesis, where LP guppies are more active feeders and may be superior invertebrate consumers, potentially occupying an equal or higher trophic position than HP guppies. However, the “specialization” and the “efficiency” hypothesis are not mutually exclusive, as guppies can both have a trophic morphology to improve acquisition of scarce high quality resources (e.g. invertebrates) and a gut that improves digestion of abundant low quality food (e.g. detritus).

In addition to generating two different predictions about the role of predation in trophic niche differentiation, these studies raise a number of other questions about trophic specialization in guppies. For example, to what extent are differences between HP and LP guppy diets driven by differences in environmental conditions (e.g. resource availability) between HP and LP sites, as opposed to trait differences between the two populations? Previous studies have relied on only 1^27^ or 2 populations of guppies^20, 21^, and have not captured the wide range of abiotic variability present in the guppy’s native range that mediates the availability of resources. Another important question is the extent to which gut contents reflect long-term vs. recent trends in diet, or, in other words, whether ingested algae or detritus is actually assimilated by these organisms. Lastly, resource availability and guppy densities are generally lower in the wet season compared with the dry season due to increased scouring events^14^, which may have significant implications for guppy foraging and diet. However, since all previous studies have focused on dry season conditions, relationships could be markedly different in the wet season when resource availability is more similar between sites, if recent conditions and availability indeed influence diet item composition.

**Figure 1:**
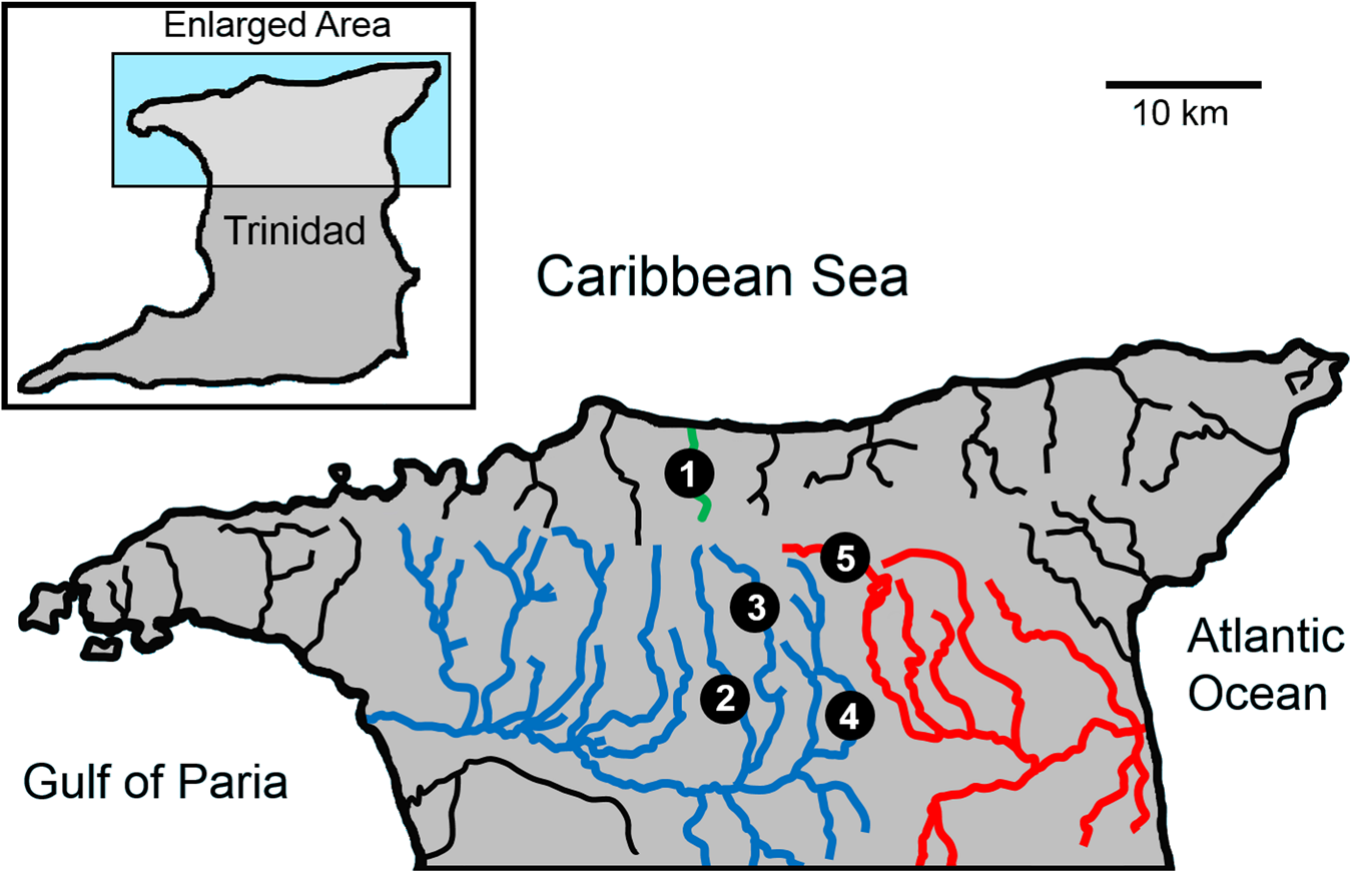
Map showing study rivers. (1) Marianne; (2) Arima; (3) Guanapo; (4) Aripo; (5) Quare. Streams constituting the Caroni drainage are in blue, the Oropuche (Quare only) in red, and the Marianne in green. Modified from El-Sabaawi *et al*. 2012.

Here, we estimate the trophic niche of guppies (i.e., their trophic position and diet) from multiple pairs of HP-LP populations in Trinidadian streams during the wet season (Fig. 1). Our study thus expands the spatial scale assessment of the influences of predation and environmental variables on the trophic niche of guppies, which is essential given the substantial differences in resource availability observed among streams with the same predation regime^17–19^. Specifically, we characterized diet and trophic position using both gut contents and stable isotopes, together with measures of environmental variables, to test predictions from the specialization vs. efficiency hypothesis of guppy diets, and to assess the role of resource abundance. Gut contents provide a ‘snapshot’ into the feeding behaviour of guppies, while stable isotope analysis allows us to infer longer-term diet assimilation over several weeks^28^, providing short and long-term information on the niche differentiation between guppy populations. Characterizing the extent and repeatability of dietary differences between guppies from HP and LP sites in natural systems can thus improve our understanding of the ecological consequences of intraspecific or phenotypic diversity.

## Results

### Stream characteristics

There were significant differences among rivers and between predation regimes for several environmental variables (Table 1, Fig. S1). However, locations with comparable fish communities (HP or LP) had similar stream characteristics across rivers, with some exceptions. HP sites generally had higher invertebrate biomass and lower epilithon ash-free dry mass (AFDM) (Table 1, Fig. S1). River of origin was also an equally important variable determining differences between sites. For example, the range in invertebrate biomass among rivers is higher than the typical difference between HP and LP sites. On the other hand, coarse particulate organic matter (CPOM/m^2^) varies more between HP and LP – being higher in LP sites - than among rivers (Table 1, Fig. S1). Detailed results are reported in Supplemental Material (S1).

**Table 1:** Characteristics of the 10 surveyed sites.

Site	Predation	Date	Fish community	Guppy density (#/m^2^)	% open canopy	Discharge (L/s)	CPOM (gDM/m^2^)	Reach width (m)	Epilithon (from pool) (gAFDM/m^2^)	FBOM (gDM/m^2^)	Inverts biomass (mgDM/m^2^)
Arima	HP	Jul-08	R, G, *Chrenicicla*, *Rhamdia*	1.01	38.4	32.0	20.5 (8.9)	32.6	3.59 (2.5)	9.92 (8.9)	971 (861)
Arima	LP	Jul-08	R, G	3.06	14.8	15.8	195 (215)	5.40	7.58 (0.5)	17.2 (15.7)	208 (266)
Aripo	HP	Jul-07	R, G, *Chrenicicla*, *Hoplias*, *Rhamdia*, *Aquidens*, *Hypostomus*/*Ancistrus*, Characidae, *Sinbranchus*	0.27	28.4	52.7	31.0 (54.2)	17.4	3.51 (1.1)	15.3 (15.0)	5517 (1867)
Aripo	LP	Jul-07	R, G	19.4	10.8	41.1	39.7 (22.2)	1.63	9.81 (6.0)	57.9 (83.3)	700 (629)
Guanapo	HP	Jul-07	R, G, *Hoplias*, *Rhamdia*, *Aquidens*, *Hypostomus*/*Ancistrus*, Characidae	2.20	18.3	na	18.6 (21.2)	na	3.36 (1.5)	9.95 (13.0)	1400 (755)
Guanapo	LP	Jul-07	R, G	5.02	11.0	32.6	40.7 (38.2)	2.37	na	21.7 (22.3)	2431 (1799)
Marianne	HP	Jul-07	R, G, *Agonostomus*, *Eleotris*, *Gobiomorus Sicydium*, *Awaous*, *Gobiesox*,	5.37	23.4	1323	112 (136)	6.61	5.39 (2.1)	16.6 (16.4)	2164.5 (1307)
Marianne	LP	Jul-07	R, G	na	12.5	1478	242 (240)	3.55	8.21 (5.3)	19.1 (22.5)	816 (476)
Quare	HP	Jul-08	R, G, *Chrenicicla*, *Hoplias*, *Rhamdia*, *Aquidens*, *Hypostomus*/*Ancistrus*, Characidae	na	48.2	57.5	17.0 (10.0)	na	9.89 (6.5)	8.67 (9.8)	415 (233)
Quare	LP	Jul-08	R, G	na	11.4	11.9	43.1 (29.3)	2.37	11.2 (4.7)	10.0 (9.8)	38.3 (47.4)

“R” is for *Rivulus hartii*, “G” is for guppy (*Poecilia reticulata*), and “na” is not available.

### Diet – Gut content

**Figure 2:**
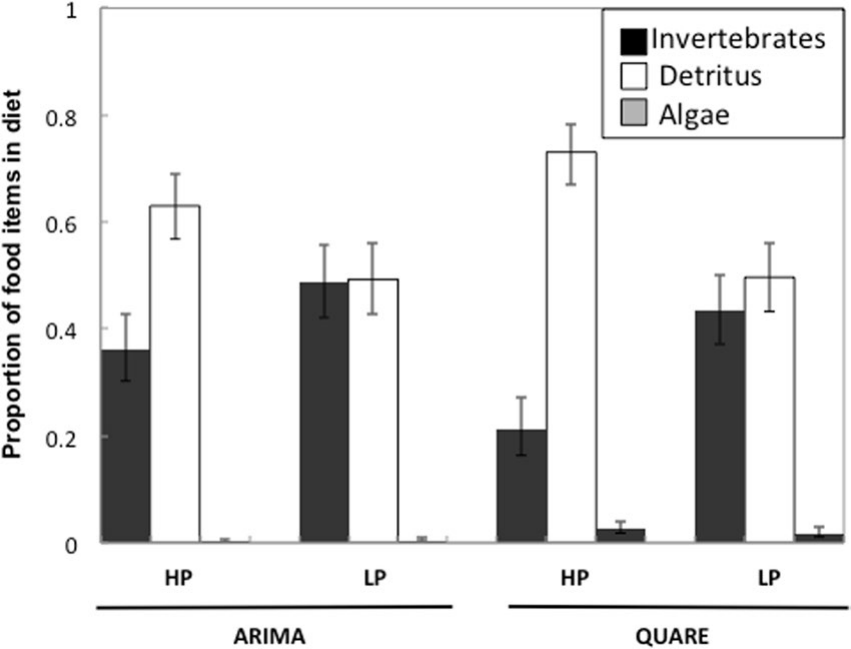
Proportion diet composition measured from the gut contents of HP and LP guppies from the Arima and Quare rivers. Data represent the estimated marginal means calculated by the GLM on arcsin transformed data, and have been back-transformed for the graphical representation. Food categories are invertebrates (dark grey), amorphous detritus (white), and algae (light grey).

Guppies from different rivers, predation regimes, and sizes had different diets. The dominant food item in guppy diets was detritus, followed by invertebrates, while algae composed a very small proportion (Fig. 2). The MANCOVA showed a significant effect of predation (F_3,46_ = 3.91; P = 0.014), river (F_3,46_ = 5.96; P = 0.002), and fish length (F_3,46_ = 11.87; P < 0.001), while the interaction between river and predation was not statistically significant (F_3,46_ = 0.98; P = 0.41).

**Figure 3:**
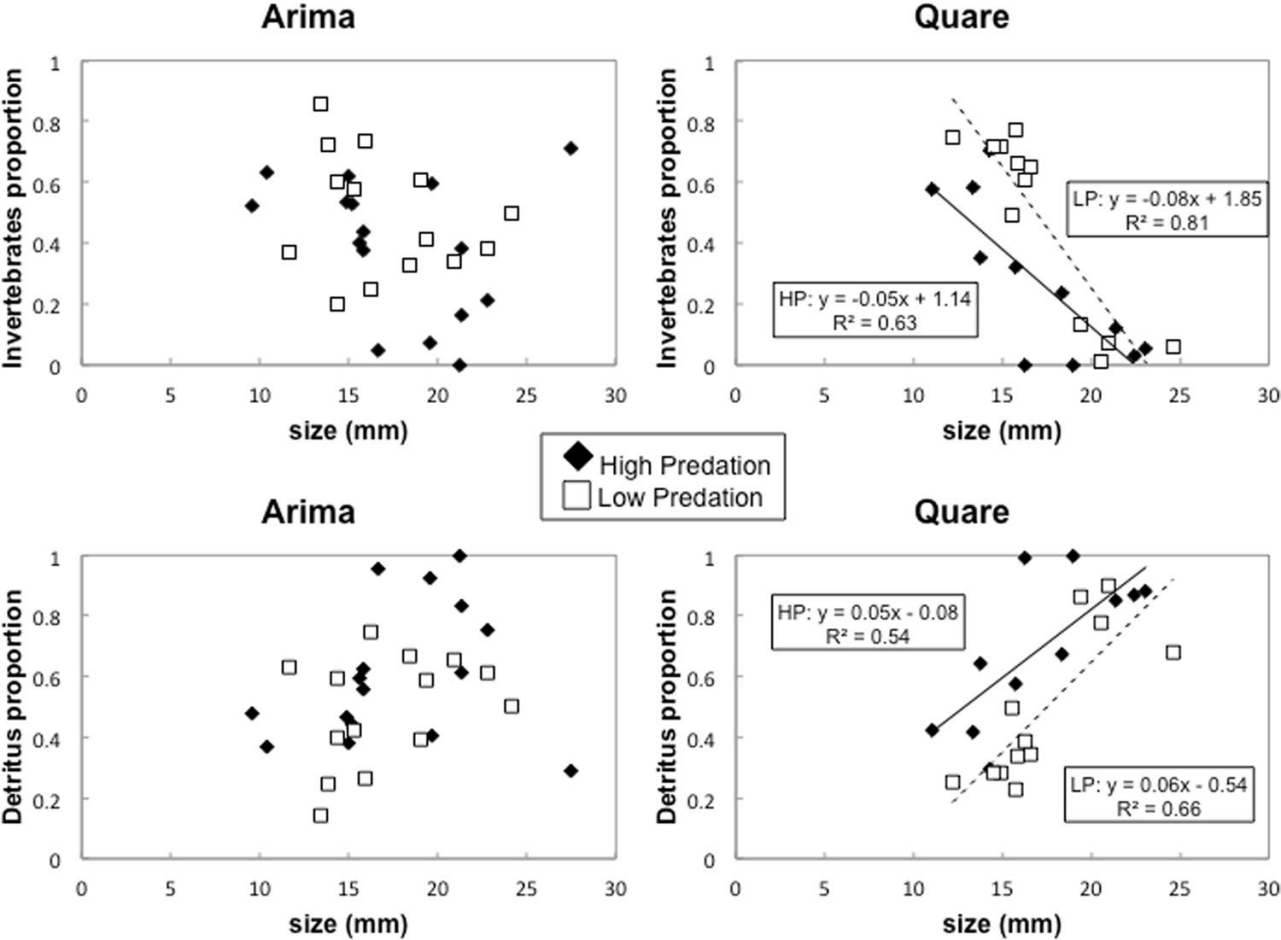
Relationship between fish length and proportion of invertebrates and detritus in diet from the Arima and Quare rivers. Only significant relationships are shown. HP guppies: filled symbols; LP guppies: open symbols.

Guppies from LP sites on the Arima and Quare rivers had significantly more invertebrates and significantly less detritus in their guts relative to guppies from HP sites (Fig. 2). Guppies in the Arima had higher invertebrates, lower detritus and lower proportions of algae in their guts compared to guppies in the Quare. The MANCOVA also showed a significant interaction between river of origin and guppy body length (F_3,46_ = 8.51; P < 0.001) for all food items (see Table S1). We further explored the causes of this heterogeneity of slopes and ran linear regressions between the proportion of the three food items and guppy body length for each river. In the Arima, there was no significant relationship between guppy length and any of the food items (all P > 0.05). In the Quare, guppies had significantly lower amounts of invertebrates in their guts with increasing size (r^2^ = 0.614, F_1,22_ = 34.96, P < 0.001), while the proportion of detritus (r^2^ = 0.491, F_1,22_ = 21.25, P < 0.001) and algae (r^2^ = 0.428, F_1,22_ = 16.48, P = 0.001) was significantly greater (Fig. 3). Trends in diet with increasing guppy size were consistent between predation communities. Finally, there was no significant relationship between the proportion of invertebrates in guppy guts and corresponding trophic position in any of the four sites.

### Trophic position

**Figure 4:**
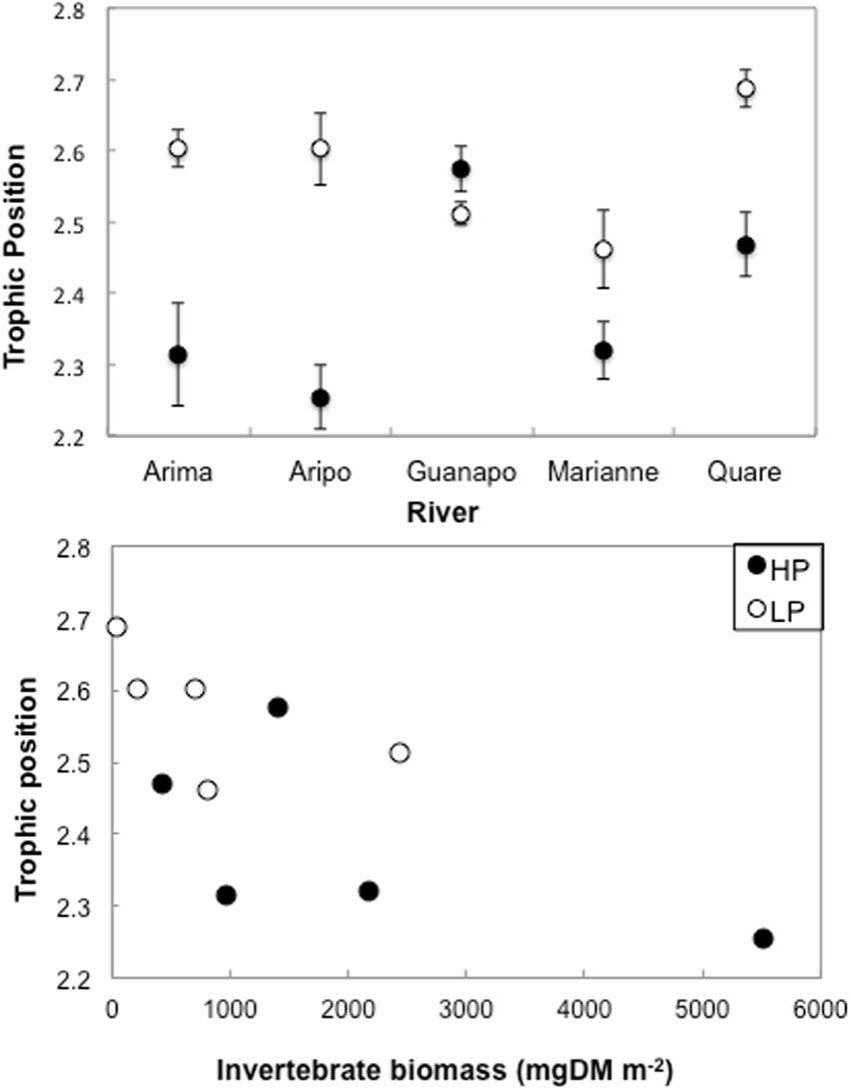
Top: Guppy trophic position across HP and LP sites in the five studied streams. Bottom: Relationship between guppy trophic position and invertebrate biomass in the five studied streams. HP guppies: filled symbols; LP guppies: open symbols. Values are averages of raw data. Error bars are ±1 standard error.

Guppies from LP sites had significantly higher trophic position than guppies from HP sites (LP = 2.63 ± SE = 0.09; HP = 2.40 ± SE = 0.06; Fig. 4), but differences were small in magnitude (less than ¼ trophic level). Sites with higher invertebrate biomass had lower trophic position (F_1,8_ = 6.94, p = 0.03; Fig. 4). A model with predation regime as a fixed effect explained variance in trophic position significantly better than one with no fixed effects (ΔAIC = 3.57, w_*i*_ = 0.86, likelihood ratio test, χ^2^ = 6.24, df = 1, p = 0.012).

Because resources are confounded with predation environment (HP having higher invertebrate biomass and lower algal stocks than LP), we used model comparisons to assess whether resources or predation risk (or a combination) better predicted variance in trophic position (Table 2). Variance in trophic position was best explained by a model with predation and invertebrate biomass as fixed effects, and removing either effect significantly reduced the explanatory power of the model (likelihood-ratio tests, removing predation term: χ^2^ = 5.02, df = 1, p = 0.025; removing invertebrate term: χ^2^ = 5.03, df = 1, p = 0.025).

**Table 2:** AIC score-based comparison of mixed models for the average trophic position of guppies from one High Predation (HP) and one Low Predation (LP) site across five different rivers.

Model Terms	Deviance	Log-Likelihood	AIC	ΔAIC	w_*i*_
Predation + Invertebrates	−24.64	12.33	−14.7	0.0	0.995
Predation	−19.63	9.81	−11.6	3.0	0.002
Invertebrates	−19.63	9.82	−11.6	3.0	0.002
Predation + Epilithon	−19.80	9.90	−9.8	4.8	0.000
Epilithon	−15.42	7.71	−7.4	7.2	0.000
No fixed Effect	−13.38	6.69	−7.4	7.3	0.000

“Predation” represents the presence or absence of effective piscivores in the site where guppies were sampled (i.e., HP vs. LP). “Epilithon” is the average AFDM from pools at a site, and “invertebrates” is the biomass of invertebrates at that site. All models include the river as a random effect to account for differences among rivers.

### Diet - Isotopic source proportions

**Figure 5:**
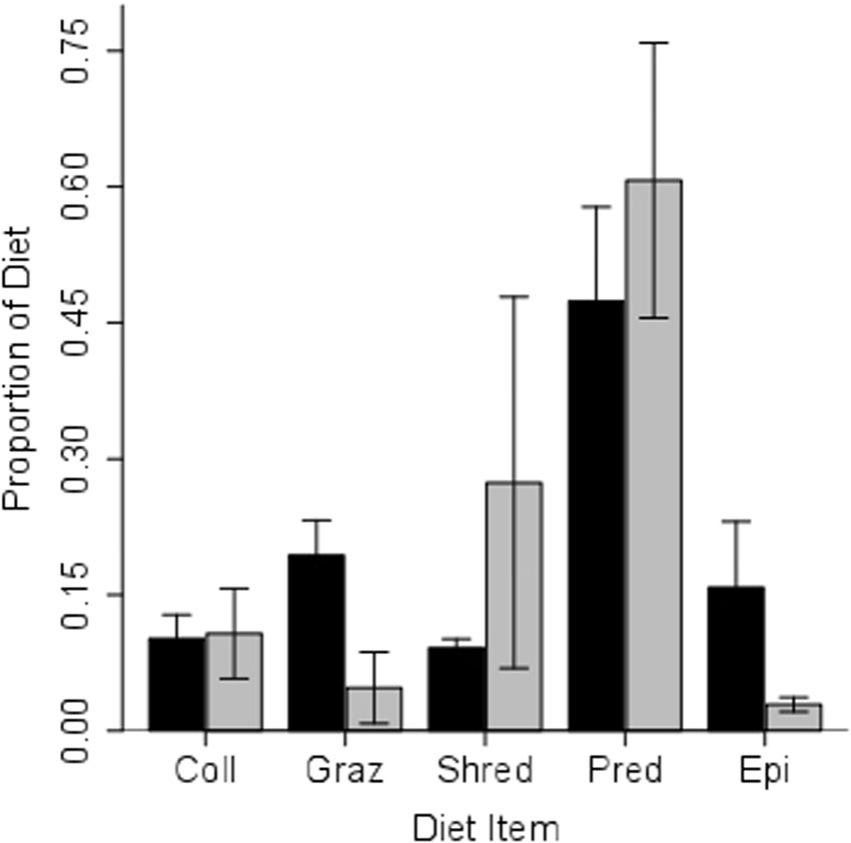
Proportion of different diet items contributing to guppy diet in HP (black) and LP (grey) sites. Data are proportions calculated from stable isotopes by the SIAR program, and error bars indicate standard errors. Pred = invertebrate predators; Coll = invertebrate collectors; Graz = invertebrate grazers; Shred = invertebrate shredders; Epi = epilithon.

The concentration-dependent isotope mixing model indicated that invertebrates are a much more important source of C and N for guppies than epilithon (Fig. 5). The concentration-dependent model, which corrects for the low C and N content of epilithon, showed that the contribution of epilithon to the assimilated diet was less than that observed in direct diet estimates, but it was significantly higher for HP (15.8% ± SE = 7.3%) than LP sites (3% ± SE = 0.8%) (Fig. 5, Supplemental Material Table S3). This difference was mostly driven by the Aripo and Quare HP sites, where the contribution of epilithon to the guppy diet was more than 30%. In the other studied sites, the proportion contribution of epilithon was on average 5% (Supplemental Material Table S4). Except for epilithon, there were no significant differences in the diets of LP and HP guppies as revealed by SIAR analysis (Fig. 5).

## Discussion

Our study across five Trinidadian streams showed that differences in predation regime and variation in resource abundance are associated with substantial intraspecific variation in the trophic niche of Trinidadian guppies, as determined by diet and stable isotope analyses. The “efficiency” hypothesis is supported by our findings that guppies in LP sites consumed and assimilated less detritus and had higher estimated trophic position than HP guppies. However, differences in trophic position between HP and LP guppies were small in magnitude (less than ¼ trophic level), and were also negatively correlated with invertebrate biomass, which is different from what previous studies conducted in the dry season had shown. We propose that LP-HP differences in diet may be sensitive to seasonal variability in resources^22^, which could lead to changes in population density that alter per capita resource availability, and ultimately the strength of intraspecific competition. We suggest further research into guppy diet and resource availability along a time series, especially focusing on the differences between dry and wet seasons, and how HP and LP guppies are differentially affected.

Gut content analyses on two of the five rivers indicated that LP fish had a higher proportion of invertebrates in their diet compared to HP guppies. The river of origin also affected diet, and we found greater difference in diet between HP and LP sites in the Quare than in the Arima. Gut content and isotopic signatures generally agree: LP guppies had slightly higher trophic positions and assimilated less algae/detritus than HP guppies. In LP sites, guppy densities are much higher and per-capita invertebrate abundance lower than in HP sites (Table 1), which suggests that there is a higher level of intraspecific competition for this high quality resource. Selective pressures might thus favour those individuals that are more efficient at feeding opportunistically on diverse types of food^27^. In HP sites, which are highly productive and have low guppy densities, HP guppies face limited competition for high-quality food and should not be resource-limited, thus selection towards feeding efficiency might be stronger in LP guppies^27, 29^. Moreover, selection on escape performance may constrain the feeding morphology of HP guppies, if trophic morphology trades off against swimming performance^29^.

In general, guppies contained larger amounts of epilithic detritus and algae in their guts than invertebrates. However, stable isotope-estimated diet contributions indicated little nutritional value is derived from epilithon and detritus food sources. Epilithon consistently represented more than 40% of the diet as detected by the volume of material in the gut, yet, on average, accounted for less than 10% of tissue C and N, suggesting assimilation of nutrients from epilithon is of limited importance. Ingestion of detritus may be incidental, a by-product of foraging for epilithon or high quality invertebrates buried in organic sediments, or a response to limited food abundance, where eating large amounts of low quality food represents a better option than eating no food at all^30^.

The relatively higher detritus ingestion and assimilation in HP guppies, as revealed by gut content and isotope results, are somehow in contrast with gut length patterns. LP guppies have longer guts than HP ones, and these differences are maintained even when diets become more similar – in the wet season^22^ and in the lab^23^. Longer guts are observed in fish that ingest low quality foods, and also in fishes with large consumption rate^31^. Such fish, referred to as “rate maximizers”, prioritize nutrient absorption from items that are easily digestible^32^. LP guppies feed at faster rates than HP guppies^27^, suggesting that they might be rate maximizers. If many of the inverts in LP sites are buried in a matrix of biofilm and fine sediments, the best way to access these foods may be to ingest large quantities of the indigestible matrix.

Our gut content data suggested that guppies from one of the rivers analysed (Quare) undergo an ontogenetic diet shift, as shown by the decreased proportion of invertebrates with guppy size. Interestingly, the ontogenetic patterns observed in gut content in the Quare were similar between predation regimes. This suggests that predators have no effect on how guppies change their feeding behaviour and microhabitat use with age, but the river of origin could have a greater effect. However, there was no indication of ontogenetic shift from the stable isotope data, likely due to the poor nutrient content of detritus, reinforcing the point that what is found in an organism’s gut does not always represent what is actually assimilated. Guppies change their microhabitat use with age^33^. Bigger guppies tend to feed on the bottom, where they might be ingesting more detritus by accident while looking for invertebrates. Young guppies, which feed at the surface, can instead eat invertebrates without having to ingest a lot of extra detritus. Adults and juveniles would thus just differ in their foraging behaviour and physiology, but not in the type of food they preferentially assimilate.

Although our findings support the efficiency hypothesis, seasonality likely also plays a prominent role in shaping guppy diets. Previous studies that supported the specialization hypothesis (that HP guppies were more carnivorous, while LP guppies were more herbivorous) were conducted in the dry season, whereas our study was conducted in the wet season. These contrasting results suggest a seasonal shift in guppy diets, where HP guppies feed more heavily on invertebrates in the dry season when invertebrate abundances are higher^22, 34^. Both guppy density and resource density decrease substantially in the wet season due to frequent high discharge events/spates^14, 34^. These changes can potentially modify interaction strength and resource use and thus altering the diet differences between LP and HP guppies. Abiotic disturbance, such as frequent high discharge events, together with lower resources and the presence of predators, could affect and limit feeding activity of guppies in HP sites more than in LP sites. These changes could result in an overall decrease in invertebrates in the diet and reduced prey selectivity in HP guppies^35^. We suggest monitoring how guppy population dynamics, resource availability, and diet preferences change along a time series, in a similar fashion as performed in a study with alewives (*Alosa pseudoharengus*)^7^. These authors sampled fish for diet and isotopic-estimated trophic position once a month for 5 months and found that trophic position changed temporally, indicating the importance of conducting a time-series sampling to catch temporal variability.

An alternative explanation is that, in the wet season, food abundance, both algae^18^ and invertebrates^34^, are often much lower, especially in LP sites. Our data indeed showed that invertebrate biomass was lower in LP sites than in HP sites and that guppy density was higher in LP sites. We have found that individual guppies from LP sites often lose weight during these periods (Reznick, pers. commun.). Experiments on guppies have shown that the ^15^N content can be 20% higher in fish receiving the same diet, but in reduced rations, in comparison with those receiving full rations^36^. This result suggests an alternative explanation for our stable isotope results, which is that the fish from our LP sites had experienced an interval of reduced food availability relative to their counterparts from HP sites and that their apparently higher trophic position is a by-product of lower food availability. This interpretation is supported by the negative correlation we found between trophic position and invertebrate biomass, where LP sites showed lower invertebrate biomass, but guppies had higher isotope-estimated trophic positions. For the three rivers for which we had density data, the biggest difference in trophic position between HP and LP is in the location at which we recorded the highest difference in guppy density (in the Aripo stream; Table 1 and Fig. 4). This could suggest that sites with very high densities had greater intraspecific competition and guppies were more food limited, potentially suffering from starvation.

Differences in environmental characteristics between HP and LP stream reaches were similar to those found in previous studies^17^. However, the correlations between environmental characteristics and predation regime make it difficult to discern if the intraspecific differences we found in guppies’ trophic niches are an effect of predation regime, the resource availability in the streams, or both. With some exceptions, HP sites all tended to have higher invertebrate biomass, and lower epilithon biomass compared to LP sites (Table 1, Fig. S1). HP sites were also bigger rivers, with lower canopy cover and higher discharge (Table 1). In a subset of the surveyed streams, guppy density was measured (Table 1) and it was always higher in LP sites. Observed patterns can be interpreted with a top-down, trophic cascade perspective. For instance, in HP sites the presence of predators keeps guppy density low, which allows invertebrate biomass to increase and basal resources to decrease. In LP sites, due to the reduced predation pressure, guppy density is higher, keeping invertebrate abundance low and relaxing the pressure on basal resources. The biomass data from our sites support this trophic cascade scenario in that guppies should be resource-limited in LP sites, becoming more omnivorous, while HP guppies should be more selective (more carnivorous). These patterns are confirmed by our dry season dataset^20^ and mesocosm experiments^21^, but not by this present study from the wet season. However, this is a simplified scenario, as these streams have high levels of omnivory and HP sites have other species of fish that can influence ecosystem processes and potentially compete for resources with guppies. The presence of these additional species in HP sites could increase interspecific competition and decrease the amount of resources available to guppies, thus affecting their diet during the wet season, when resources are less abundant.

This study is one of the few to report evidences of intraspecific trophic differences in a riverine fish, which appeared to be linked to predation pressure and environmental characteristics. Intraspecific niche differences have been observed in other species, when different populations adapt to different habitats or resource types and abundances (e.g. Anolis lizards^37^, Galapagos finches^38^). Many examples of such polymorphisms are found in lacustrine species of fish (e.g. sticklebacks, salmonids, cichlids^5, 39, 40^), where different trophic habits are associated with phenotypic differences, but such studies are very rare for riverine fish (but see ref. 2). Guppies from LP and HP localities also show morphological differences^27^, but they are not as pronounced as those found in some of the lacustrine species cited above. In guppies, predator avoidance may represent a stronger selective pressure than foraging efficiency for morphological characteristics^27^. Also, even if HP and LP sites differ in their environmental characteristics, in lakes resource type and availability could be more contrasting between habitats (e.g. benthic vs limnetic), thus driving more pronounced trophic differences and polymorphism. Lastly, abiotic disturbance, such as that caused by seasonality, could be more important in driving differences in niche use in riverine species than in lacustrine ones, where spatial variability might instead play a predominant role.

Intraspecific niche differences like those observed in guppies can have important implications in eco-evolutionary feedbacks, as shown for landlocked and anadromous alewives populations^7^. These feedbacks can take place when an organism modifies its environment, and in this way changes the selective pressure it experiences, further modifying its impact on the environment, and thus generating a feedback loop^14^. In the case of guppies, the predation intensity can affect, both directly and indirectly, the amount of resources that are available to guppies, thus favouring specific diet choices. Guppies’ prey selection can thus affect the resources available in the environment, thus further changing guppies’ prey choices. This can have consequences in the functioning of the ecosystem and ecological communities^21, 41^ and can even lead to speciation or adaptive radiation, as shown for other species^42^.

In conclusion, this study, which was the first natural abundance isotope study conducted with guppies from their natural environment, helps us understand the factors that can affect guppy trophic niches and, more in general, the causes of intraspecific trophic differentiation. Guppies showed intraspecific differences across a large spatial scale in their trophic position and diet preferences as a function of predation regime and environmental characteristics. Observed differences among predation regimes are compatible with the hypothesis that LP guppies are more efficient invertebrate predators rather than being specialized on basal resources. Moreover, the present study suggests that seasonality could play an important role in determining guppy trophic niches by relaxing some of the selective pressures present during the dry season, potentially altering the system to one controlled by abiotic forces. Lastly, studies that aim to find repeatable patterns should incorporate populations from several rivers, and the factors causing differences between rivers should be investigated into greater details.

## Materials and Methods

We sampled five different rivers in the Northern Range of the Caribbean island of Trinidad (Fig. 1). Three of the rivers (Arima, Aripo, and Guanapo) were located in the South slope of the range and were part of the Caroni drainage, one of them (Quare) in the East Slope belonging to the Oropuche drainage, and one of them (Marianne) in the North Slope. In each river we sampled a pair of reaches: one HP and one LP site. HP and LP sites were generally located along an altitudinal gradient, with HP being more downstream and LP more upstream. All of the HP sites, save the Marianne, included some of the most common guppy predators such as *Crenicichla sp*. and *Hoplias malabaricus* ^43^. The Marianne had a different ichthyofauna than the other rivers, and the main predators of guppies here were *Eleotris pisonis*, *Agonostomus monticola*, and *Gobiomorus dormitor* (Table 1). Shrimp are present in the East and North slopes streams, but are absent from the South slope.

We conducted this study during the wet season: 3 rivers (Aripo, Guanapo, Marianne) were sampled between 6 July and 14 August 2007, and the other 2 (Arima, Quare) on 12–26 July 2008. All samples were collected from each site over one or occasionally two sampling dates. In each site, we sampled a stream reach of approximately 100–200 m of length, which comprised at least 3 separate pools and riffles. In each of the three pools and riffles we sampled epilithon (EPI) and invertebrate biomass, since these resources most likely reflect food items of guppies. We also measured several environmental variables to characterize the sites including guppy density, fine (FBOM) and coarse (CPOM) benthic organic matter, percent open canopy, and discharge. A detailed description of sampling methodology is reported in the Supplemental Material S1.

To assess differences in diet between predation regimes, guppies of both sexes were collected in the 2008 summer from two focal rivers, the Quare and Arima, for diet analyses. From each, we examined contents from the stomach and a small part of the foregut, and measured a subsample of the total area occupied by the most abundant food items: invertebrates, algae (diatoms and filamentous), and detritus, as described elsewhere^16^. Invertebrates were identified to the lowest possible taxonomic level, usually the family category^44^.

Guppies of both sexes were also collected with potential diet items from all sites to estimate the relative contribution of each item to guppy diets using stable isotope mixing models. To estimate the stable isotopic composition of guppy tissues, we captured a minimum of 17 and a maximum of 48 guppies (mean and median = 26) of all size classes per site. Viscera were removed and each fish was dried to constant weight at 55 °C.

The most abundant invertebrate taxa were collected, and were allowed to incubate for gut clearance. Several individuals of each taxon were pooled for SIA analysis. Epilithon, a mixture of algae, detritus, and microbial biofilm, was collected from rocks using a Loeb sampler^45^. Prior to stable isotope analysis, all samples were dried at 50–60 °C and were then ground to fine powder with a mortar and pestle. Approximately 1 mg of each sample (~3 mg for epilithon samples) was weighed and loaded into tin capsules. Samples were analysed using a Finnigan Delta C mass spectrometer connected to a Carlo Erba 1500 CHN analyser at the Stable Isotopes Ecology Laboratory at the University of Georgia (Athens, Georgia, USA). Isotopic ratios (heavy isotope/light isotope) are expressed in the typical δ notation as parts per thousand deviation from international standards, which is atmospheric nitrogen for δ^15^N and Pee Dee belemnite limestone for δ^13^C.

From these data, we calculated guppy trophic position using the formula proposed by^46, 47^:

1 $${\rm{TP}}=[({{\rm{\delta }}}^{15}{{\rm{N}}}_{{\rm{gup}}}-{{\rm{\delta }}}^{15}{{\rm{N}}}_{{\rm{base}}})/{\rm{\Delta }}]+{\rm{\lambda }}$$

where: Δ is the fractionation factor that we chose equal to 2.9‰ (from ref. 48; and λ ( = 1) is the trophic position of the baseline, δ^15^N_base_ is the signature of the baseline, which is a mixture of two invertebrate primary consumers, chosen as described below.

Because δ^15^N of basal resources varies significantly in time and space, several studies have recommended using the δ^15^N of a primary consumer as the baseline for trophic position measurements^46, 47^. As a baseline, we chose to average the δ^15^N of two primary consumers collected at each site: *Psephenus sp*., a grazing water penny, and *Phylloicus sp*., a shredding caddisfly. These two taxa were among the most common primary consumers that were present in almost all of our sites, and overall had among the lowest δ^15^N signatures. In a few sites, we were not able to collect one of these two common taxa. In these cases, we calculated an average difference for all sites between *Psephenus* and *Phylloicus* and used it to calculate the δ^15^N of the missing taxa. Consequently, all of our baseline values were site-specific. We chose to use the average value between a shredder and a grazer, since they represent the baselines for allochthonous and autochthonous pathways respectively, both of which are likely to be important in our streams^49^.

We performed Univariate ANOVAs to assess differences in environmental variables (e.g., epilithon and invertebrate biomass, coarse benthic organic material) between predation regimes and rivers. We ran a multivariate analysis of covariance (MANCOVA) to test for differences in guppy gut content across rivers and predation regimes. Percentages of invertebrates, detritus, and algae (diatoms and filamentous) were the dependent variables of the MANCOVA, and river (Arima and Quare), predation level (HP, LP), and their interaction were included as fixed factors. We used fish standard length as a covariate. Because the interaction between river and fish length was significant, we included it in the model. For these two rivers, we also examined the linear relationship between guppy trophic position (estimated from δ^15^N values as explained above) and percentages of invertebrates in the guts using a linear regression.

We analysed environmental influences on trophic position using linear models. The environmental variables that we included in the model were invertebrate and epilithon (AFDM) biomass, which were those that had a significant effect on trophic position (Table S2). To assess the role of predation regime (HP vs. LP), we compared two linear mixed models for trophic position: one with predation regime as a fixed effect and river as a random effect, and another with only river as a random effect. Because both invertebrate and epilithon abundance are confounded with predation environment (see Table 1 with environmental data), we compared candidate models with Akaike’s information criterion (AIC) to assess whether changes in the resources (i.e. epilithon and invertebrate biomass), changes in predation (i.e., HP vs. LP), or both, better explained variance in trophic position among sites. Guppy standard length did not have a significant effect on trophic position, as tested by linear regression, and it was not included in the model (Supplemental Material S2).

In order to estimate the proportion contribution of each food item to guppy diet, we employed a Bayesian mixing model using the *SIAR* R package, which incorporates multiple dietary sources and generates solutions as probability distributions of the different sources^38^. It also incorporates the variability of the sources, the end members (consumers), and the fractionation factors. For guppy discrimination values, we chose to use 2.9 ± 0.4‰ for δ^15^N and 1.1 ± 0.1‰ for δ^13^C^48^. We performed normalization of lipid content for the guppies’ δ^13^C signatures using a linear equation from^50^:

2 $${{\rm{\delta }}}^{13}{{\rm{C}}}_{\mathrm{lipid} \mbox{-} \mathrm{normalized}}={{\rm{\delta }}}^{13}{{\rm{C}}}_{{\rm{bulk}}}-3.32+0.99\times {\rm{C}}:{\rm{N}}$$

SIAR allows the use of many dietary sources, however, like other mixing models (e.g. IsoSource), its performance decreases with an increasing number of sources. Thus, we reduced the number of sources by including only the most abundant taxa found in guppy diets (present in at least 10 guppy guts), which we then pooled into functional feeding groups, which differed in isotopic values. The sources we chose for the SIAR were: invertebrate collectors, grazers, shredders, predators and epilithon (EPI). We used the concentration-dependent SIAR model, which includes the percent carbon (C) and nitrogen (N) content of the food items^51^ and aims to recreate the actual diet on a dry weight basis.

To assess divergence in diet between HP and LP sites, we modelled the effect of predation regime on the SIAR-estimated diet contributions of each food item at each sampling site. In these models, we modelled the site-level mean percent contribution of each of the five possible diet items (*i*.*e*., predators, shredders, collectors, grazers, shredders, epilithon) as our response variable. We then constructed two linear mixed models for the proportion of that item in the diet: a null model with only river as a random effect to describe non-independence of HP and LP sites in the same river, and an alternative model with both predation regime as a fixed effect and river as a random effect. χ^2^ model comparisons were used to determine if the predation explained a significant proportion of the total variance in the proportion of the diet composed of each item.

Data were arcsin (for percentages) and log transformed to meet normal distribution assumptions when necessary and significance set at p < 0.05. All statistical analyses were carried out using SPSSStatistics 18.0 and the R statistical environment^52^.

### Ethics statements

Fish were collected and handled with the approval of the University of California Riverside IACUC AUP (no. A-20080008) and conforming to the legislation in Trinidad. All experimental protocols were approved by the University of California Riverside IACUC AUP (no. A-20080008).

## Electronic supplementary material

Supplementary Information
