# Supplementary material for: Population variation in the trophic niche of the Trinidadian guppy from different predation regimes
Source: Sci Rep. 2017 Jul 18;7:5770. doi: 10.1038/s41598-017-06163-6 (PMC5515894; doi:10.1038/s41598-017-06163-6)
Supplement: Supplementary file 1 — Supplementary Information [file 41598_2017_6163_MOESM1_ESM.docx]

**Supplementary Information**

**Population variation in the trophic niche of the Trinidadian guppy from different predation regimes**

**Authors: Eugenia Zandonà^1,7^, Christopher Dalton^2^, Rana W. El-Sabaawi^2,8^, Jason L. Howard^1,9^, Michael C. Marshall^3,^, Susan S. Kilham^1^, David N. Reznick^4^, Joseph Travis^5^, Tyler J. Kohler^6,10^, Alexander S. Flecker^2^, Steven A. Thomas^6^, Catherine M. Pringle^3^**

**Supplemental Information 1 – Environmental characteristics sampling methodologies and differences between LP and HP sites**

*Description of environmental variables sampling methodology*

Algal standing stocks were sampled by scraping 5 rocks from 3 pools and 3 riffles at each site. We assessed algal biomass by measuring the ash-free dry mass (AFDM) of a predetermined subsample of the scraped slurry that was filtered on a 25 mm pre-combusted and weighed GFF (at 450^o^C for 2 hours). The filter was then dried at 105^o^C until it reached a constant weight, it was then weighed and oxidized in a muffle furnace at 500^o^C and reweighed. The AFDM weight is equal to the weight of the dried material plus the filter, minus the filter weight, minus the weight of the remaining ashes after the oxidation. The AFDM weight is then divided by the subsampled area to obtain the AFDM/m^2^. AFDM represents a proxy for algal biomass, but it does not distinguish between algal material and other organic matter, such as fungi and bacteria; furthermore it also includes the biomass of senescent material ([Steinman, et al. 2007](#_ENREF_6)).

Benthic invertebrates were sampled in 3 pools and 3 riffle habitats in each site using a Hess sampler ([Hess 1941](#_ENREF_3)). Samples were then immediately stored in 95% ethanol solution (which is generally diluted to 70% due to the little water in the sample). Back in the lab, samples were stained with rose bengal for at least 24 hours and then separated using 2 sieves (1mm and 250 μm). Only the > 1mm and between 1 mm and 250 μm fractions were picked and identified at the lowest taxonomic level (generally Family or Genus) following ([Perez 1996](#_ENREF_5)) and ([Merritt, et al. 2007](#_ENREF_4)). The <250 μm fraction was not used for this analysis, as we were not interested in estimating meiofauna. Samples that had more than 100-150 invertebrates were subsampled using a plankton splitter. Invertebrates were counted and identified using a dissecting scope. Invertebrate biomass was calculated using length-mass regression equations from the literature ([Benke, et al. 1999](#_ENREF_1)) and from estimates of Trinidadian specimens (T. Heatherly unpubl. data).

Fine benthic organic matter (FBOM) was collected in 3 pools and 3 riffles in 2007 and 2 pools and 2 riffles in 2008 in each site. We collected FBOM using a sampling corer and graduated bucket following the procedure described in ([Wallace, et al. 2007](#_ENREF_7)). All the material collected was passed through nested sieves of 63 μm, 250 μm, and 1000 μm and everything that was retained by the sieves, including what passed through the smaller sieve, was stored in bags. Back in the lab, the material collected by each sieve was then dried at 50^o^C until it reached a constant weight and then ashed at 500^o^C. AFDM/m^2^ for each size fraction was calculated following the formulas in ([Wallace, et al. 2007](#_ENREF_7)) and then summed up to obtain the total FBOM biomass of the whole sample.

Coarse particulate organic matter (CPOM) is mostly composed of allochthonous material (mainly leaves). CPOM biomass was assessed by randomly tossing a pvc frame of known area in the stream and collecting all material within the frame by hand. The material was then dried at 50^o^C for at least 48 hours or until completely dry, and subsequently weighed. We collected CPOM in 3 pools and 3 riffles in 2007 and 3 random spots in 2008.

Percent open canopy was measured using a hemispherical densiometer. Pool width and depth were measured at transects across the pool, then averaged. Pool widths measurements are all wetted width. Discharge was measured using the midsection method ([Gore 2007](#_ENREF_2)).

We performed Univariate ANOVAs to assess differences in environmental variables between predation regimes and rivers. For each ANOVA, the dependent variables we used were benthic invertebrate biomass/m^2^, epilithon AFDM/m^2^, CPOM/m^2^, and FBOM/m^2^ (only the 63-250 μm size fraction). In the model we also included the interaction between predation and river.

**Environmental sampling results**

There were significant differences in environmental variables between predation sites, between rivers, and in some cases between years of sampling. Overall, locations with comparable fish communities (HP vs. LP) had similar stream characteristics across rivers, with some exceptions. HP sites generally had higher invertebrate biomass, lower epilithon ash-free dry mass (AFDM), lower CPOM, and a non-significant tendency toward lower FBOM (Table 1). River of origin was also an equally important variable determining differences between sites, especially for the benthic invertebrates, FBOM, and epilithon AFDM. Invertebrate standing stocks were lower in the 2008 survey than they were in the 2007 survey.

Univariate ANOVA showed that predation (F_1,48_=41.11; P<0.001), river of origin (F_4,48_=19.66; P<0.001), and the interaction of these two factors (F_4,48_=5.85; P=0.001) all had significant effects on benthic invertebrate biomass found in the stream. Benthic invertebrate biomass was higher in HP than LP sites for all rivers but the Guanapo, which showed the opposite pattern (Fig. S1). Invertebrate standing stocks were highly variable between rivers. Post-hoc tests (LSD) showed that benthic invertebrate biomass in the 3 rivers sampled in 2007 did not differ between each other, while the Arima and Quare, sampled in 2008, had significantly less biomass than that measured in rivers sampled in 2007. The Quare river had the lowest invertebrate biomass.

FBOM/m^2^ stock was not affected by predation regime (F_1,36_=2.55; P=0.119), but differed between rivers (F_4,36_=4.78; P=0.003). The interaction between predation and river of origin was also not significant in regards to FBOM (F_4,36_=0.36; P=0.832). The Quare river had the lowest FBOM, while the Aripo had the highest (Fig. 2). CPOM/m^2^ stock was higher in LP than in HP sites (F_1,33_=4.32; P=0.045). We did not find a significant effect of river of origin (F_4,33_=2.12; P=0.100) or of the interaction between predation and river (F_4,33_=0.12; P=0.974). The Marianne had the highest CPOM/m^2^ and was significantly different from the Aripo and Guanapo, which had the lowest CPOM/m2 standing stocks (Fig. S1).

In all five rivers, LP sites had higher epilithon AFDM/m^2^ than HP sites (Univariate ANOVA, predation: F_1,47_=19.20; P<0.001). River of origin also had a significant effect on the epilithon AFDM/m^2^ (F_4,47_=5.21; P=0.001), but there was no interaction between predation regime and river (F_4,47_=1.33; P=0.272). The Quare had significantly greater epilithon AFDM/m^2^ than the other 5 rivers. The Arima had the lowest standing stock of epilithon (AFDM/m^2^) and was significantly different from the Quare and Turure, but not from the Aripo and Marianne (Fig. s1). In sites where we had density estimates, guppy density in LP sites appeared to be generally higher than densities in HP sites. We did not test these observations statistically due to lack of sample size.

**Figure S1**

Biological characteristics of the 12 sites sampled in Trinidad. Top left: logarithm of the benthic invertebrate biomass (mg/m^2^). Top right: logarithm of the epilithon (EPI) ash-free dry mass (AFDM) per meter square (g/m^2^). Bottom left: logarithm of the benthic organic matter (BOM) ash-free dry mass (AFDM) per meter square (g/m^2^). Bottom right: logarithm of the coarse particulate organic matter (CPOM or leaf litter) (mg/m^2^). The Turure river was not included in the invertebrates, BOM, and CPOM analyses, as we could not sample its low predation site. The Guanapo river was not included in the EPI analysis as the samples were lost. High predation sites are in dark grey and low predation sites in light grey. Values are estimated marginal means calculated by the Univariate ANOVA. Error bars are ± 1 SE.


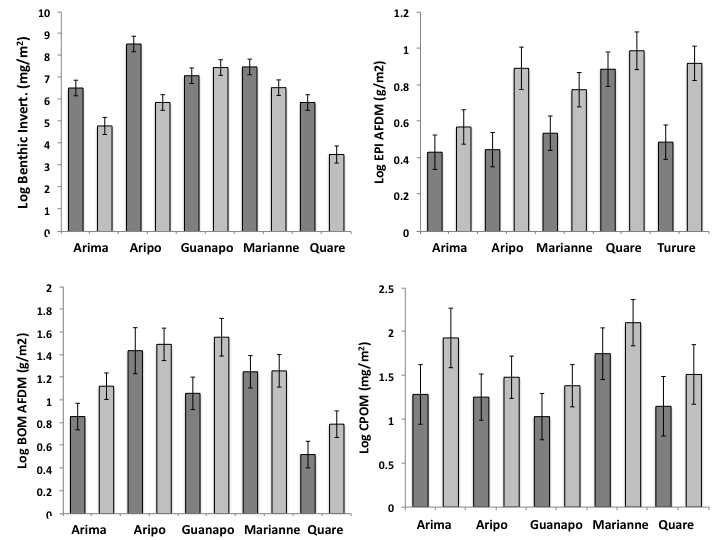


**Supplemental Information 2 – Gut and Trophic position analysis**

**Table S1.** Univariate ANCOVA results summarizing the effects of predation and river on the proportion of invertebrates, detritus, and algae in the guppy diet from stomach content analysis. SS=type-III sums of squares; df=degrees of freedom; MS=mean squares.

| **Univariate ANCOVA** | | | | | |
| --- | --- | --- | --- | --- | --- |
| **Source** | **SS** | **df** | **MS** | **F-ratio** | **P-value** |
| *Invertebrates* |  |  |  |  |  |
| Predation | 0.45 | 1 | 0.45 | 8.01 | 0.007 |
| River | 0.49 | 1 | 0.49 | 8.80 | 0.005 |
| Fish Length | 1.54 | 1 | 1.54 | 27.41 | <0.001 |
| Predation*River | 0.04 | 1 | 0.04 | 0.75 | 0.390 |
| River*Fish Length | 0.65 | 1 | 0.65 | 11.63 | 0.001 |
| Error | 2.69 | 48 | 0.056 |  |  |
| *Detritus* |  |  |  |  |  |
| Predation | 0.48 | 1 | 0.48 | 9.36 | 0.004 |
| River | 0.21 | 1 | 0.21 | 4.15 | 0.047 |
| Fish Length | 0.95 | 1 | 0.95 | 18.38 | <0.001 |
| Predation*River | 0.03 | 1 | 0.03 | 0.68 | 0.414 |
| River*Fish Length | 0.27 | 1 | 0.27 | 5.22 | 0.027 |
| Error | 2.49 | 48 | 0.052 |  |  |
| *Algae* |  |  |  |  |  |
| Predation | 0.00 | 1 | 0.00 | 0.04 | 0.842 |
| River | 0.12 | 1 | 0.12 | 11.48 | 0.001 |
| Fish Length | 0.16 | 1 | 0.16 | 15.05 | <0.001 |
| Predation*River | 0.01 | 1 | 0.01 | 0.82 | 0.369 |
| River*Fish Length | 0.19 | 1 | 0.19 | 17.66 | <0.001 |
| Error | 0.52 | 48 | 0.01 |  |  |

**Table S2:** Models for the effects on trophic position of each of the six environmental characteristics measured. Presented here are the parameter estimates (and standard errors) for the effect of each variable on trophic position, and model comparison to a model with no fixed effect. The presented p-value represents the significance of the variance explained by the model with the environmental measurement relative to a model with no fixed effects.

| Measurement | Parameter | Std. Error | ΔAIC | w*_i_* | p |
| --- | --- | --- | --- | --- | --- |
| Canopy Cover (%) | -0.67 | 0.50 | 0.09 | 0.49 | 0.167 |
| Guppy Density (# × m^-2^ × 100) | 0.22 | 1.05 | 2.11 | 0.26 | 1.000 |
| CPOM (gDM × m^-2^ × 1000) | -0.8 | 0.9 | 1.03 | 0.37 | 0.324 |
| Algae (mg × m^-2^ × 100) | 3.00 | 0.67 | **0.00** | **0.76** | **0.038** |
| BOM (g AFDM × m^-2^ × 100) | -0.0011 | 0.0041 | 2.08 | 0.26 | 1.000 |
| Invert Biomass (g × m^-2^ × 1000) | -0.0677 | 0.0295 | **0.00** | **0.81** | **0.027** |

**Figure S2**

Bi-plots of guppy trophic position (y-axis) and δ^13^C signatures (x-axis) for the 5 rivers sampled. Each data point represents an individual guppy. HP guppies are indicated with filled, while LP in open symbols.


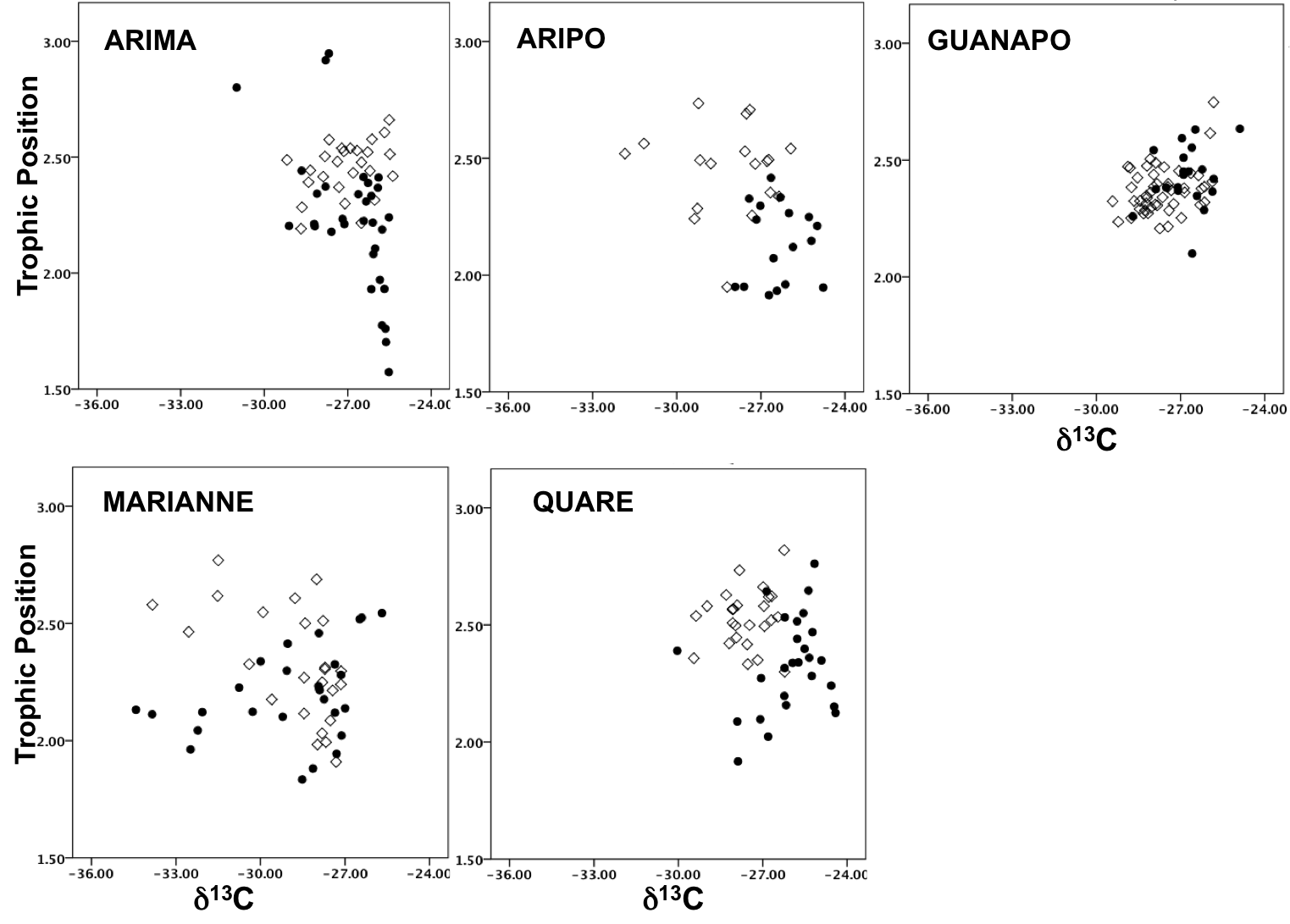


**Figure S3**

Relationship between trophic position and proportion of invertebrates found in the guts in the Arima and Quare rivers. HP guppies are indicated with filled, while LP in open symbols. No significant relationship was found.


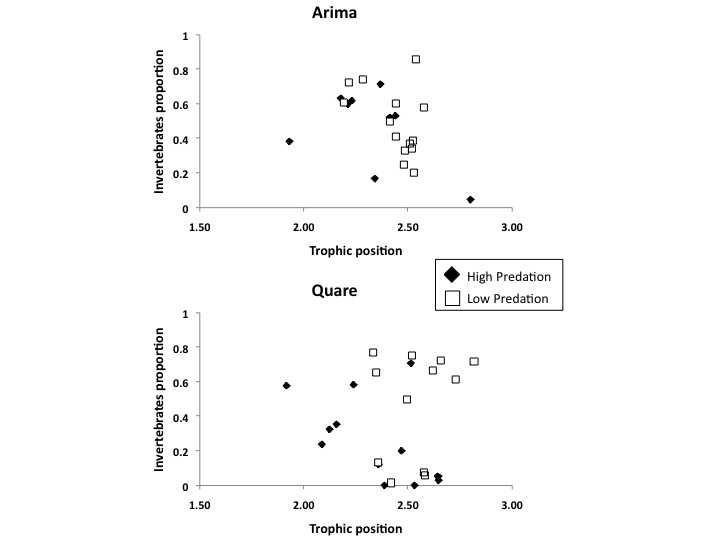


**Incorporating length into the trophic position analysis**

To assess the potential influence of length on trophic position, we ran a simple linear regression of trophic position vs. length (t = 1.50; P=0.14). We also compared a mixed model for trophic position with length as a fixed effect and river as a random effect to one with just a river fixed effect (Chi-sq = 2.38; P=0.12). We thus decided to not incorporate length in our models for trophic position: there appears to be no effect of length on trophic position and factoring in length does not eliminate the effect of predation on trophic position.

**Table S3:** Estimated differences in isotope-estimated diets between LP and HP populations across four rivers. Shown are the results of analysis with linear mixed models for the proportion of each diet item in guppy tissues at each site, with predation regime as a fixed effect and river as a random effect. Shown are estimated differences (and standard errors) between HP and LP populations, AIC scores for models with and without predation, and the difference in AIC score between the model with predator minus the AIC score of that with only river as a random effect. *χ*^2^ and *p* values from a *χ*^2^ model comparison of the linear models with and without predation risk p-value are shown.

|  | Pred. Effect on Diet | | AIC Scores | | ΔAIC  (w/-w/o pred) | *χ*^2^ | *P* |
| --- | --- | --- | --- | --- | --- | --- | --- |
|  | HP - LP | Std Err. | w/o pred | w/ pred |  |  |  |
| Collectors | + 0.02 | 0.06 | 76.2 | 78 | 1.8 | 0.2 | 0.665 |
| Grazers | + 0.13 | 0.09 | 89 | 88.8 | -0.2 | 2.2 | 0.138 |
| Shredders | - 0.01 | 0.06 | 83.9 | 85.9 | 2 | 0 | 0.909 |
| Predators | + 0.01 | 0.06 | 77.1 | 79.1 | 2 | 0 | 0.911 |
| Epilithon | + 0.14 | 0.06 | 85.1 | 82.5 | -2.6 | 4.5 | **0.034** |

**Table S4.** Proportion contribution of each food item to the guppy diet estimated by the SIAR model for each studied site. Numbers represent percent of diet composed of each source.

|  | **Collector** | **Grazer** | **Shredder** | **Predator** | **Epilithon** |
| --- | --- | --- | --- | --- | --- |
| **Arima HP** | 7 | 8 | 7 | 75 | 3 |
| **Arima LP** | 6 | 1 | 3 | 87 | 2 |
| **Aripo HP** | 9 | 18 | 11 | 27 | 34 |
| **Aripo LP** | 31 | NA | 13 | 50 | 6 |
| **Guanapo HP** | 11 | 30 | 9 | 44 | 7 |
| **Guanapo LP** | 4 | 1 | 88 | 6 | 1 |
| **Marianne HP** | 4 | 25 | NA | 69 | 2 |
| **Marianne LP** | 8 | 17 | NA | 73 | 3 |
| **Quare HP** | 20 | 15 | 9 | 23 | 33 |
| **Quare LP** | 6 | 1 | 4 | 85 | 3 |

**References**

Benke, A. C., Huryn, A. D., Smock, L. A. and Wallace, J. B. 1999. Length-mass relationships for freshwater macroinvertebrates in North America with particular reference to the southeastern United States. - Journal of the North American Benthological Society 18: 308-343.

Gore, J. A. 2007. Discharge measurements and streamfolw analysis. - In: Hauer, F. R. and Lamberti, G. A. (eds.), Methods in Stream Ecology. Academic Press, pp. 51-78.

Hess, A. D. 1941. New limnological sampling equipment. - Limnological Society of America Special Publication 6: 1-5.

Merritt, R. W., Cummins, K. W. and Berg, M. B. 2007. An Introduction to the Aquatic Insects of North America. 4th Edition. - Kendall/Hunt Publishing Company.

Perez, G. R. 1996. Guia para el estudio de los macroinvertebrados acuaticos del Departimento de Antioquia. - Universidad de Antioquia.

Steinman, A. D., G.A. Lamberti and P.R.Leavitt. 2007. Biomass and Pigments of Benthic Algae. - In: Hauer, F. R. and G.A.Lamberti (eds.), Methods in Stream Ecology. Academic Press, pp. 357-379.

Wallace, J. B., Hutchens, J. J. and Grubaugh, J. W. 2007. Transport and storage of FPOM. - In: Hauer, F. R. and Lamberti, G. A. (eds.), Methods in stream ecology. Academic Press, pp. 249-271.
